# Sprouty4 Is an Endogenous Negative Modulator of TrkA Signaling and Neuronal Differentiation Induced by NGF

**DOI:** 10.1371/journal.pone.0032087

**Published:** 2012-02-23

**Authors:** Fernando C. Alsina, Dolores Irala, Paula A. Fontanet, Francisco J. Hita, Fernanda Ledda, Gustavo Paratcha

**Affiliations:** 1 Division of Molecular and Cellular Neuroscience, Institute of Cellular Biology and Neuroscience Prof. Dr. E. De Robertis (IBCN)-CONICET, School of Medicine, University of Buenos Aires (UBA), Buenos Aires, Argentina; 2 Laboratory of Molecular and Cellular Neuroscience, Department of Neuroscience, Karolinska Institute, Stockholm, Sweden; Hertie Institute for Clinical Brain Research and German Center for Neurodegenerative Diseases, Germany

## Abstract

The Sprouty (Spry) family of proteins represents endogenous regulators of downstream signaling pathways induced by receptor tyrosine kinases (RTKs). Using real time PCR, we detect a significant increase in the expression of *Spry4* mRNA in response to NGF, indicating that Spry4 could modulate intracellular signaling pathways and biological processes induced by NGF and its receptor TrkA. In this work, we demonstrate that overexpression of wild-type Spry4 causes a significant reduction in MAPK and Rac1 activation and neurite outgrowth induced by NGF. At molecular level, our findings indicate that ectopic expression of a mutated form of Spry4 (Y53A), in which a conserved tyrosine residue was replaced, fail to block both TrkA-mediated Erk/MAPK activation and neurite outgrowth induced by NGF, suggesting that an intact tyrosine 53 site is required for the inhibitory effect of Spry4 on NGF signaling. Downregulation of Spry4 using small interference RNA knockdown experiments potentiates PC12 cell differentiation and MAPK activation in response to NGF. Together, these findings establish a new physiological mechanism through which Spry4 regulates neurite outgrowth reducing not only the MAPK pathway but also restricting Rac1 activation in response to NGF.

## Introduction

The best-characterized member of the neurotrophins is nerve growth factor (NGF), which supports the differentiation and survival of specific populations of sensory, sympathetic, and central nervous system neurons via the activation of its receptor tyrosine kinase, TrkA. Once activated, TrkA receptors trigger intracellular signal transduction cascades, including those mediated by Ras/Mitogen-Activated Protein Kinase (MAPK), PI3-kinase (PI3K)/Akt, PLC and other pathways regulated by Rho family of small GTPases. Activation of these pathways allows NGF to regulate neuronal differentiation and survival [1], [2]. At the same time, receptor tyrosine kinases also trigger a complex chain of molecular events, named negative signaling, that reduce the strength and duration of positive signals to finally modulate the cellular physiology. Previous studies have revealed that receptor tyrosine kinase signaling is tightly regulated through the coordinated action of several protein inhibitors that function at multiple levels of the signaling cascade and at different time-points after receptor engagement [3]. While positive signaling effectors are relatively well understood, signaling attenuation still remains elusive [3], [4].

During the last years, the Sprouty (Spry) family of proteins (Sprouty1–4) has emerged as negative signaling regulators of many trophic factors [5]. Sprouty was first described as an inhibitor of fibroblast growth factor (FGF)-stimulated tracheal branching during *Drosophila* development [6]. Subsequent studies revealed that mammalian genome contain four *Sprouty* genes encoding proteins of 32–34 kDa. It has been reported that trophic factors regulate the activity of Sprouty inducing its expression and promoting the phosphorylation of Sprouty proteins on critical tyrosine residues [5].

The emerging picture from Sprouty's studies indicates that they specifically inhibit Ras-Raf-MAPK pathway activated by a wide range of trophic factors, including FGF [6], [7], BDNF (Brain-Derived Neurotrophic Factor) [8], GDNF (Glial cell line-Derived Neurotrophic Factor) [9], PDGF (Platelet-Derived Growth Factor) [10] and VEGF (Vascular Epithelial Growth Factor) [11], but do not affect MAPK activated by EGF (Epidermal Growth Factor) [12]. The molecular mechanism through which Sprouty antagonizes MAPK remains unclear, and may depend on the cellular context or the RTK involved. Interestingly, Sprouty2 was reported to potentiate biological effects induced by EGF, inhibiting epidermal growth factor receptor ubiquitination and downregulation [13]–[15]. These findings suggest that the role of Sprouty in RTK signaling is still controversial, because they may regulate RTK signaling in a negative or positive manner.

The regulation of neuronal physiology and the development of the nervous system require a tight spatial and temporal control, which is partly achieved by negative feedback loops, involving the expression of inhibitory proteins that counteract neurotrophic factor receptor signaling. Despite of the essential contribution of NGF for neuronal development and function, the molecular mechanisms that control NGF-induced TrkA signaling are not totally understood. Previous work has shown that expression of Sprotuy4, but not Sprouty1 or 2, could be induced after NGF treatment of PC12 neuronal cells [10], [12]. However, despite this evidence, the functional contribution of Sprouty4 to NGF signaling and biology has not been explored, yet. In view of this, we decided to investigate whether Sprouty4 might play a prominent role controlling TrkA signaling and biological responses to NGF.

In this study, we determine that Sprouty4 is a TrkA-induced gene that restricts Erk/MAPK pathway and inhibits Rac1, but not Akt, activation in response to NGF. At the molecular level, we identify the Tyr-53 of Sprouty4 as an essential determinant for this inhibitory activity. Here, we also show that Sprouty4 negatively regulates NGF-induced neuronal differentiation of PC12 cells and primary sensory neurons.

## Results

### NGF induces Sprouty4 expression in neuronal cells

**Figure 1:**
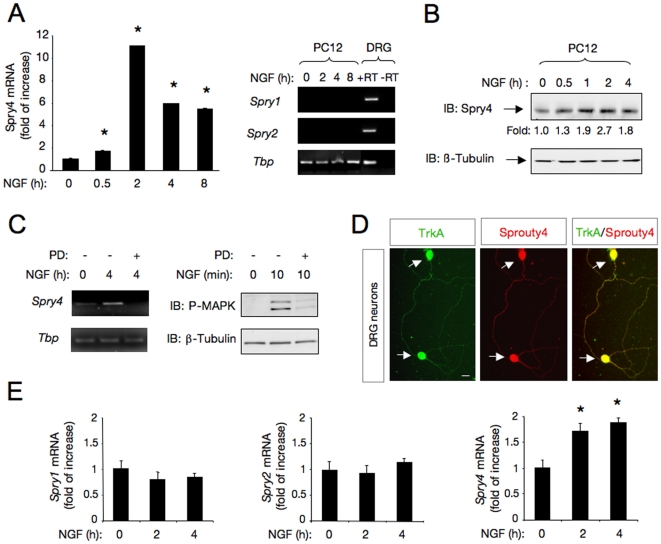
NGF signaling induces Sprouty4 in neuronal cells. A) Left panel, quantitative analysis of S*prouty4* mRNA expression by real-time PCR in PC12 cells treated with NGF (50 ng/ml) during the indicated times. The levels of *Sprouty4 (Spry4)* mRNA were normalized using the expression of the housekeeping gene *Tbp* (TATA binding protein). Shown are averages ± SD of triplicate determinations. *p<0.001 versus control (Ctrl) group (one-way ANOVA followed by Dunnett's test). Right panel, expression of *Spry1* and *Spry2* mRNAs examined by RT-PCR (35 cycles) in DRG and PC12 cells treated with NGF (50 ng/ml) for different time-points. Expression of the housekeeping gene *Tbp* was used as loading control. The experiment was repeated two times with similar results. B) Western blot analysis of Sprouty4 expression in PC12 cells treated with NGF (50 ng/ml). Reprobing control was done with antibodies against -tubulin. Fold change relative to -tubulin is indicated. C) Left panel, expression of *Spry*4 mRNA examined by semiquantitative RT-PCR (27 cycles) in PC12 cells treated with the specific MEK inhibitor PD98059 (50 M) and stimulated with NGF (50 ng/ml) as indicated. Expression of the housekeeping gene *Tbp* was used as loading control. Right panel shows a control experiment performed in parallel to verify the inhibitory activity of PD98059 on MAPK pathway. PD98059 activity was controlled measuring MAPK activation by immunoblotting of PC12 cells stimulated for 10 min with NGF (50 ng/ml). Reprobing control was done with antibodies against -tubulin. D) Colocalization of Sprouty4 and TrkA in DRG dissociated neurons obtained from E14.5 rat embryos detected by immunofluorescence. Scale bars: 10 m.E) Quantitative analysis of S*pry1,* S*pry2* and S*pry4* mRNA expression by real-time PCR in DRG neurons, treated with NGF (50 ng/ml) during the indicated times. The *Sproutys* mRNA levels were normalized using the expression of the housekeeping gene *Tbp*. Shown are averages ± SD of triplicate determinations. *p<0.01 versus control (Ctrl) group (one-way ANOVA followed by Dunnett's test).

Recent studies on the mechanisms that restrict RTK signaling revealed the importance of negative-feedback control of receptor function as a mechanism to ensure signaling thresholds compatible with a physiological response. Based on this concept, we analyzed whether Spry4 is induced on neuronal cells after NGF stimulation. Using real-time PCR, we examined the expression pattern of *Spry4* mRNA in PC12 cells in response to NGF. This quantitative analysis revealed a significant induction of *Spry4* mRNA in this neuronal cell line responsive to NGF (Fig. 1A, left panel). The *Spry4* mRNA induction was additionally observed by semiquantitative RT-PCR (Supplementary Fig. S1). In contrast, neither *Spry1* nor *Spry2* mRNAs were detected by RT-PCR in PC12 cells in the absence or presence of NGF (Fig. 1A, right panel). Spry4 protein up-regulation was additionally confirmed by immunoblotting of PC12 cell lysates treated with NGF (Fig. 1B).

Previous work showed that Erk/MAPK activation pathway by mitogenic stimuli induces Spry4 in cultured cells [12]. To examine this possibility, PC12 cells were incubated in the presence or absence of a specific inhibitor of Erk/MAPK pathway, PD98059, before stimulation with NGF. The mRNA level of Spry4 was determined by semiquantitative RT-PCR from samples prepared 4 h after ligand stimulation, and compared with cells that were not treated with PD98059. As shown in Figure 1C, left panel, pharmacological inhibition of the MAPK pathway with PD98059, abrogated the NGF-induced *Spry4* mRNA expression, suggesting that an intact MAPK pathway is required for Spry4 up-regulation in response to NGF. The inhibitory activity of PD98059 was controlled in parallel, measuring MAPK activation by immunoblotting of PC12 cell extracts stimulated for 10 min with NGF (Fig. 1C, right panel).

To address whether Spry4 may play a physiological role in NGF/TrkA-driven dorsal root ganglion (DRG) neuron development, we examined whether Spry4 and the NGF receptor, TrkA, are co-expressed in DRG neurons, a neuronal subpopulation highly responsive to NGF. Primary dissociated DRG neurons obtained from E14.5 rats were assessed by immunofluorescence using anti-Spry4 and anti-TrkA antibodies. The staining revealed a striking co-localization of Spry4 and TrkA expression in DRG neurons (Fig. 1D).

Using real-time PCR, we examined the pattern of mRNA expression of *Spry1, Spry2 and Spry4* in response to NGF in primary DRG neurons. In these experiments, we only detected a significant increase in the expression of *Spry4* mRNA in response to NGF (Fig. 1E). Thus, the specific induction of *Spry4* mRNA detected in DRG neuronal cultures treated with NGF indicates that Spry4 may play a physiological role in NGF/TrkA-driven DRG neuron development and function.

### Sprouty4 specifically inhibits NGF-induced Erk/MAPK and Rac1 activation

**Figure 2:**
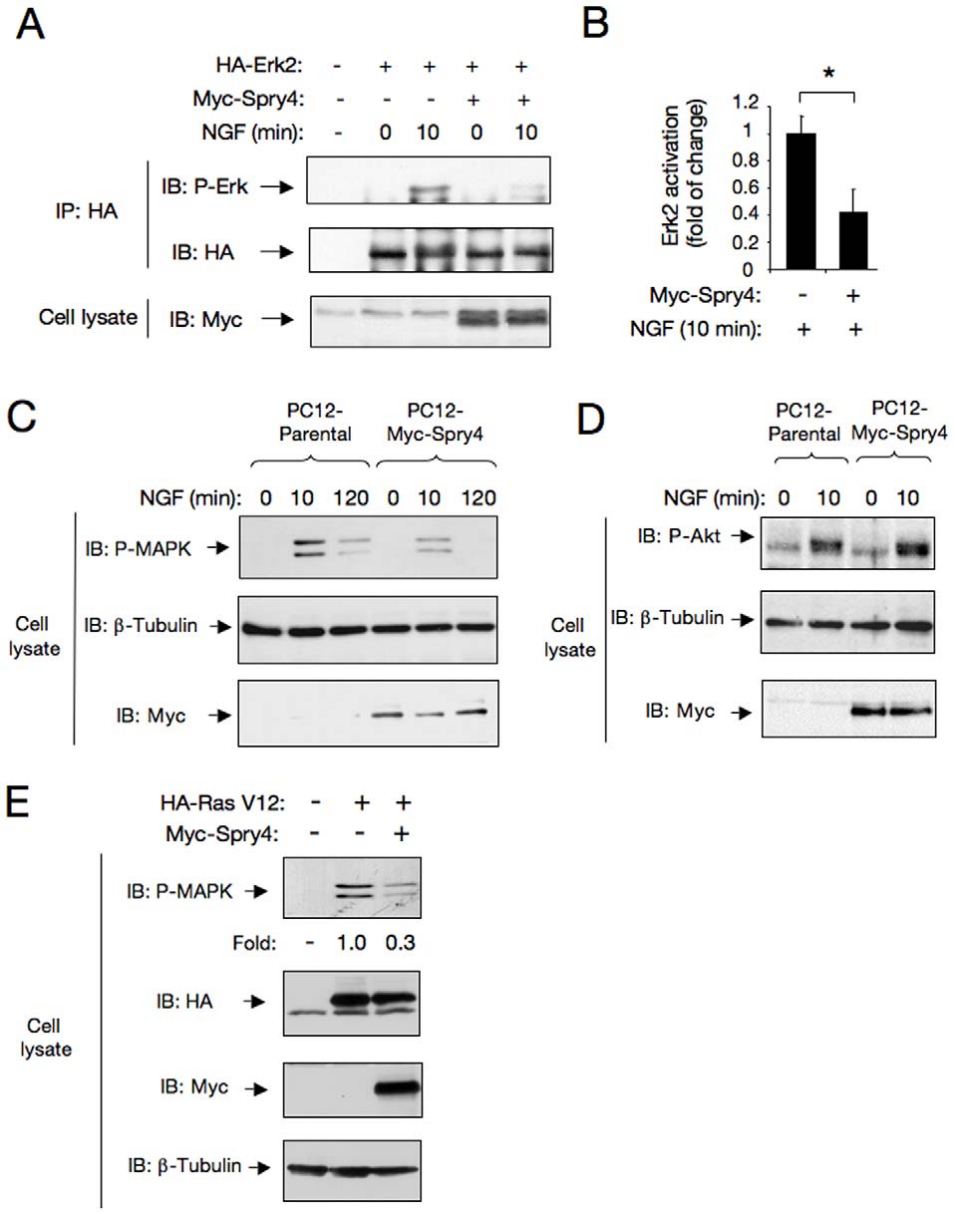
Sprouty4 restricts Erk/MAPK activation in response to NGF. A) Erk2/MAPK activation (Phospho-Erk2/MAPK) was evaluated by transient transfection of HA-tagged Erk2 plasmid with a control or a Myc-tagged Sprouty4 vector into PC12 cells. After 36 h cells were serum-starved and stimulated with or without NGF for 10 min. The level of Erk2 activation (P-Erk2) was evaluated by HA-immunoprecipitation followed by immunoblotting with a specific antibody that recognizes the phosphorylated forms of ERK/MAPK. Reprobing of the same blot with anti-HA and anti-Myc antibodies is shown. B) Histogram shows quantification of Erk2/MAPK activation. Results are presented as averages ± SD from three independent experiments. *p<0.05 (Student's t test). C) Erk/MAPK activation (P-MAPK) in cell lysates of parental and PC12-Spry4 (Clon S2) cells treated with NGF (50 ng/ml) and detected by immunoblot (IB). Reprobing of the same blot with anti–tubulin and anti-Myc antibodies is shown. D) Akt activation (P-Akt) in cell lysates of parental and stable transfected PC12-Spry4 (Clon S2) cells treated with NGF (50 ng/ml) and detected by IB. Reprobing of the same blot with anti–tubulin and anti-Myc antibodies is shown. E) Ligand-independent Erk/MAPK activation (P-MAPK) was evaluated by transient transfection of HA-tagged Ras-V12 plasmid (constitutively active Ras) together with a control or a Myc-Sprouty4 vector into PC12 cells. After 36 h, cells were serum-starved and the levels of Erk/MAPK activation were evaluated by immunoblot with a specific antibody that recognizes the phosphorylated forms of ERK/MAPK. Reprobing of the same blot with anti-HA, anti-Myc and anti–tubulin antibodies is shown. Fold change relative to the level of constitutive active HA-Ras V12 is indicated.

To establish whether Spry4 could modulate NGF/TrkA signaling, we investigated its role on different intracellular signaling pathways induced by NGF. TrkA receptors transmit intracellular signals through several of the canonical pathways activated by other RTKs, including the Ras/MAP kinase, PI3K/Akt, PLC and other signaling pathways controlled by Rac1 GTPases. To determine whether Spry4 could regulate the Erk/MAPK pathway downstream of TrkA, we transiently transfected HA-tagged Erk2/MAPK plasmid with control or Myc-tagged Spry4 vector into PC12 cells. After 36 h, cells were serum-starved and stimulated with or without NGF for 10 min. The level of Erk2 activation was evaluated by HA-immunoprecipitation followed by immunoblotting with a specific antibody against phosphorylated Erk1 and Erk2/MAPK. PC12 cells expressing Spry4 showed a significant reduction in Erk2/MAPK activation compared to control cells (Fig. 2A and 2B). Similar results were obtained by comparing NGF-dependent endogenous MAPK activation in lysates prepared from parental PC12 cells and clones overexpressing Spry4 (PC12-Spry4 cells) (Fig. 2C). On the contrary, Spry4 could not reduce NGF-induced Akt activation in response to NGF in the same clones (Fig. 2D).

In order to explore at which level Spry4 could restrict MAPK activation, we co-expressed a constitutively active V12-Ras together with Spry4 in PC12 cells. In this experiment Spry4 was able to reduce ligand-independent MAPK activation by constitutively active V12-Ras, indicating that Spry4 impairs MAPK activation acting downstream Ras (Fig. 2E).

**Figure 3:**
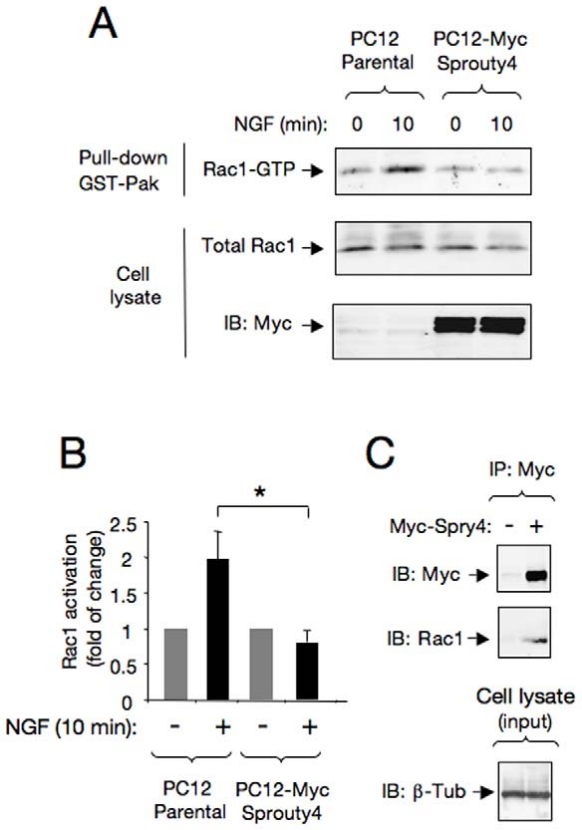
Sprouty4 restricts Rac1 activation in response to NGF. A) Rac1 activation (Rac1-GTP) in cell lysates of parental PC12 cells and clones overexpressing Spry4 (PC12-Spry4 cells) stimulated with NGF (50 ng/ml) for 10 min. Rac1 activation was assessed by GST-Pak-CRIB pull-down assay, followed by immunoblot (IB) with anti-Rac1 antibodies. The bottom panels show total Rac1 and Myc-tagged Sprouty4 levels present in cell lysates. B) Histogram shows quantification of Rac1 activation. Results are presented as averages ± SEM from three independent experiments. *p<0.05 (Student's t test). C) Co-immunoprecipitation between endogenous Rac1 and Myc-tagged Sprouty4 transiently transfected in Cos cells. Mock and Myc-tagged Sprouty4 transfected cell lysates were immunoprecipitated (IP) with anti-Myc antibodies. Immunoprecipitated proteins were resolved by SDS-PAGE and the filters immunoblotted with anti-Rac1 antibodies. The bottom panel shows equal level of -tubulin in both sample inputs.

Rho-like GTPases, including RhoA, Rac1 and Cdc42 are critical proteins in transducing neurotrophin signals to the actin cytoskeleton [16]. In particular, it has been described that neurite outgrowth induced by NGF/TrkA is mediated by Rac1 activation [17], [18]. Since Spry2 has previously been reported to regulate cell migration of rat intestinal epithelial cells by specifically inhibiting the activation of Rac1, but not Cdc42 GTPases [19], we evaluated whether Spry4 could regulate NGF-induced Rac1 activation in PC12 and PC12-Spry4 cells stimulated with NGF (Fig. 3A). The level of active Rac1 (Rac1-GTP) was measured by using an affinity-precipitation assay with the GST-tagged Rac1-GTP interacting binding domain of Pak. As it has been previously described, Rac1 was activated in PC12 cells after 10 min of NGF stimulation. However, the activity of Rac1 was significantly reduced in PC12-Spry4 cells, indicating that Spry4 negatively regulates Rac1 activation triggered by NGF (Fig. 3A and 3B, and Supplementary Fig. S2). Indeed, the specific co-immunoprecipitation between endogenous Rac1 and Myc-tagged Spry4 reported in Fig. 3C, suggests that these two molecules might directly associate and additionally support its functional interaction.

### Sprouty4 inhibits neuronal differentiation of PC12 cells and DRG neurons in response to NGF

**Figure 4:**
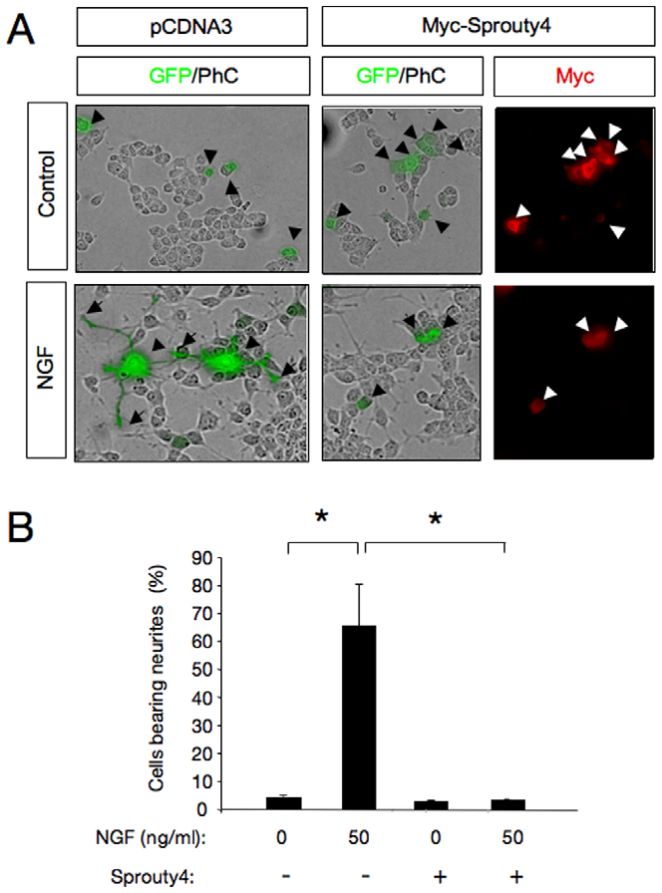
Sprouty4 inhibits neuronal differentiation of PC12 cells in response to NGF. A) Photomicrographs show PC12 cells transfected with control or Myc-tagged Sprouty4 plasmid together with a GFP expression vector. After 72 h of NGF treatment (50 ng/ml), cells were fixed and stained with anti-Myc antibodies. Arrowheads indicate neuronal cell bodies and arrows denote neurite tips. Scale bar: 10 m. B) Histogram shows quantification of the relative number of GFP positive PC12 cells bearing neurites longer than 1.5 cell body diameters after 72 h of treatment with NGF. The results are presented as averages SD of a representative experiment performed in triplicate. *p<0.001 (ANOVA followed by Student Newman Keuls). The experiment was repeated three times with similar results.

As it is known that TrkA signaling contributes to NGF-mediated survival and differentiation of various neuronal populations, we next investigated whether Spry4 could modulate neurite outgrowth of neuroblast-like PC12 cells in response to NGF. For this purpose, PC12 cells were transfected with control or Myc-tagged Spry4 plasmid in combination with an enhanced green fluorescent protein (GFP) expression vector. Cells were maintained in the presence of NGF for 72 h and stained with anti-myc antibodies to confirm that all GFP-positive cells express Spry4. To exclude the possibility that the effects seen overexpressing Spry4 were due to apoptosis, the morphology of the nuclei was controlled using the nuclear staining DAPI. Interestingly, overexpression of Spry4 inhibited NGF-induced PC12 neurite outgrowth. A significant reduction in cells bearing neurites was observed after transfection with Myc-tagged Spry4 plasmid (Fig. 4A and 4B, and Supplementary Fig. S3). This result demonstrates that Spry4 restricts NGF-mediated morphological differentiation of PC12 neuronal cells.

In agreement with the control of Rac1 activity by Spry4 described above (Fig. 3), ectopic expression of a dominant negative Rac1 (Rac1-DN) was able to mimic the action of Spry4, attenuating NGF-induced PC12 cell differentiation (Supplementary Fig. S4).

**Figure 5:**
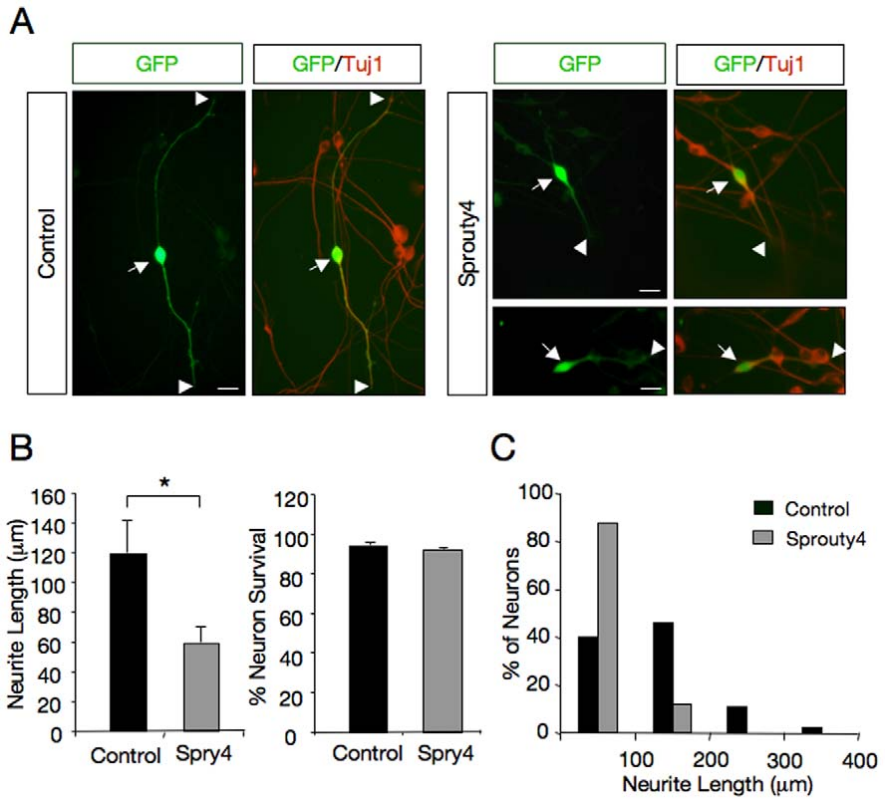
Sprouty4 inhibits neurite outgrowth of dorsal root ganglion neurons (DRG) in response to NGF. A) Dissociated DRG neurons transfected with GFP in the absence (Control) or in the presence of an excess of Myc-tagged Sprouty4 (Spry4) construct were cultured with NGF (50 ng/ml). After 36 h in culture, neurons were fixed and stained with anti–tubulin antibodies. Scale bar represents 20 m. Arrows indicate neuronal cell bodies and arrowheads denote neurite tips. B) Left panel, histogram showing the inhibition of neurite outgrowth in DRG neurons by exogenous expression of Sprouty4. The results are averages SEM of a representative experiment measured in six wells per experimental group, *, p<0.05 (Student's t test). The experiment was repeated three times with similar results. Right panel, histogram showing the survival of DRG neurons by exogenous expression of Sprouty4. Neuronal survival was evaluated using the nuclear staining DAPI. GFP-positive neurons containing fragmented or condensed nuclear staining were scored as apoptotic cells. The results are averages SEM of a representative experiment performed in triplicate. C) Histogram shows the distribution of neurons carrying neurites in different length categories after transfection with GFP in the absence (Control) or in the presence of Myc-tagged Sprouty4. A total of 43 control- and 40 Sprouty4-transfected neurons from a representative assay were evaluated. Note the noticeable shift to the left of the distribution of neurons that received the Sprouty4 construct.

Next, we also examined the role of Spry4 on neurite outgrowth induced by NGF in primary DRG neurons. To this end, dissociated DRG (E14.5) neurons were transfected with control or Myc-tagged Spry4 construct together with a GFP expression vector. In agreement with the results obtained in PC12 cells, neurite outgrowth stimulated by NGF was significantly reduced in cells transfected with Myc-Spry4 (neurite length ± SEM in m, control: 119.4±21.0; Myc-tagged Spry4: 59.6±4.9, n = 6; *p<0.05) (Fig. 5A, 5B, left histogram and 5C). No differences were observed in NGF-promoted neuron survival between the two groups (Fig. 5B, right histogram). Data expressed as average values ± SEM are as follow: control: 94.1±4.2; Spry4 vector: 92.0±3.4, n = 3; p>0.05). These results indicate that inhibition of TrkA signaling by Spry4 specifically restrict neuronal differentiation but not survival.

### Sprouty4 Tyr-53 is essential for its inhibitory activity

**Figure 6:**
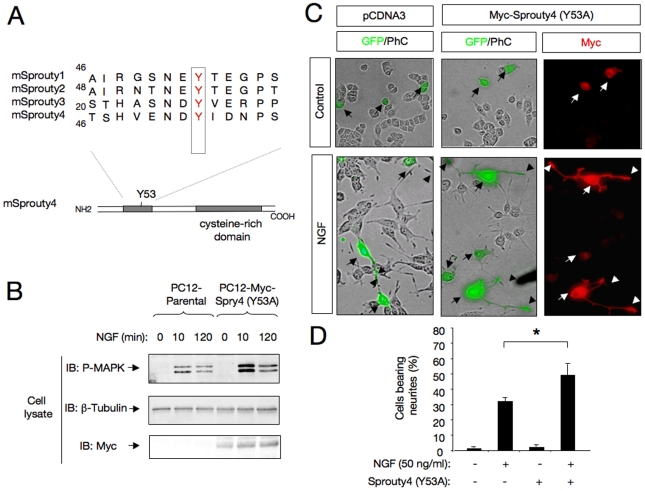
Sprouty4 Y53A mutant loses its ability to block both Erk/MAPK pathway and neurite outgrowth of PC12 cells in response to NGF. A) Schematic representation of the domain structures of mammalian Sprouty family members. The figure shows an amino acid alignment of a conserved motif located at the N-terminal half of the molecule. There, a conserved tyrosine residue (Y) is indicated in red. The conserved C-terminal cysteine-rich domain is also shown. B) Erk/MAPK activation (P-MAPK) in cell lysates of parental and PC12-Myc-Spry4 Y53A cells treated with NGF (50 ng/ml) and detected by IB with a specific antibody that recognizes the phosphorylated forms of ERK/MAPK. Reprobing of the same blot with anti–tubulin and anti-Myc antibodies is shown. The experiment was repeated two times with similar results. C) Photomicrographs show PC12 cells transfected with control or Myc-tagged Sprouty4 Y53A mutant together with a GFP expression vector. After 72 h of NGF treatment, the cells were fixed and stained with anti-Myc. Arrows indicate neuronal cell bodies and arrowheads denote neurite tips. Scale bar: 10 m. D) The histogram shows the quantification of the relative number of GFP positive PC12 cells bearing neurites longer than 1.5 cell body diameters after 72 h of treatment with NGF. The results are presented as averages SD of a representative experiment performed in triplicate. *p<0.05 (ANOVA followed by Student Newman Keuls). The experiment was repeated three times with similar results.

To further understand the mechanisms through which Spry4 inhibits NGF signaling, we generated a PC12 cell line expressing a mutant Spry4 in which tyrosine 53 was substituted by Alanine (PC12-Spry4-Y53A cells). Interestingly, this tyrosine is conserved among the four Sprouty family members (see Fig. 6A) and it was reported to have a dominant negative effect enhancing trophic factor-dependent Erk/MAPK activation [12]. To assess the role of this point mutation, parental PC12 and PC12-Spry4-Y53A cells were stimulated with NGF for 10 and 120 min and then Erk/MAPK activation was measured using anti-phospho-ERK antibodies. As shown in Fig. 6B, PC12 cells expressing Spry4-Y53A showed a substantial increase in Erk/MAPK activation compared to control cells, indicating that this Y53A substitution generates a non-functional Spry4 mutant, that loses its ability to block NGF-dependent Erk/MAPK pathway.

Then, we investigate the effect of Spry4 Y53A mutant on NGF-mediated neurite outgrowth. For this purpose, PC12 cells were transiently transfected with control or Myc-tagged Sprouty4 Y53A plasmid in combination with an enhanced GFP. Cells were maintained in the presence of NGF for 72 h and stained with anti-myc antibodies to confirm that all GFP-positive cells express Spry4. Overexpression of Spry4 Y53A could not inhibit NGF-promoted neurite outgrowth, but rather slightly enhanced it (Fig. 6C, 6D and Supplementary Fig. S3). However, no tyrosine phosphorylated Spry4 could be detected after treatment with saturating concentrations of NGF (Supplementary Fig. S5). Taken together, these assays indicate that Tyr-53 in itself, but not its phosphorylation, is required for Spry4 biological function. Our results are in agreement with previous data showing that Spry4 could not be tyrosine phosphorylated upon treatment with serum and different growth factors including EGF, PDGF, FGF [20].

### Spry4 knockdown potentiates PC12 cell differentiation in response to NGF

**Figure 7:**
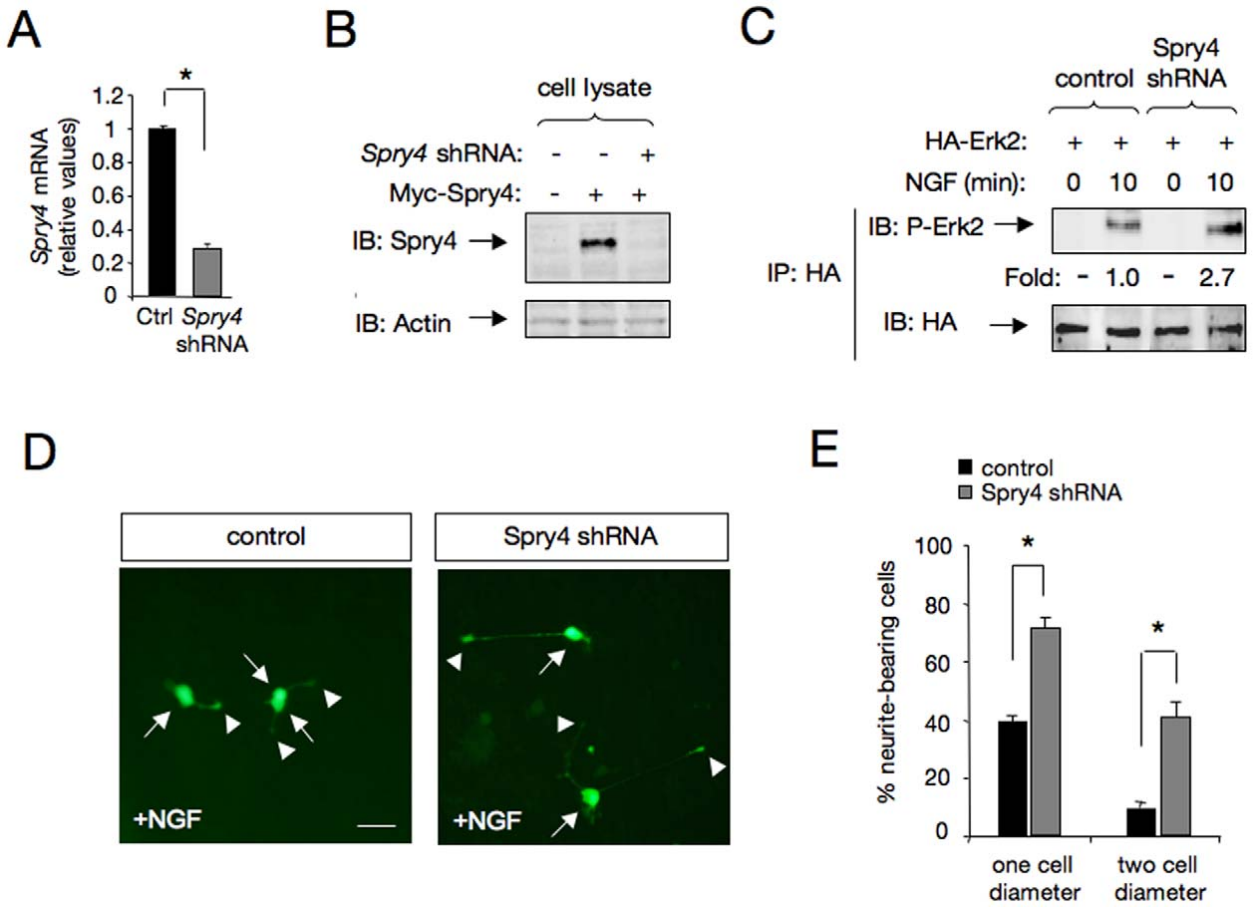
Knockdown of Sprouty4 potentiates MAPK activation and PC12 cell differentiation in response to NGF. A) *Spry4* mRNA levels were analyzed by real-time PCR in PC12 cells transfected with scrambled (control, Ctrl) or *Spry4* shRNA constructs. Transfected cells were enriched by puromycin treatment in order to increase the population of cells expressing scrambled or *Spry4* shRNA constructs. Quantitative analysis is shown as averages SD of triplicate determinations. The levels of Spry4 mRNA were normalized using the expression of the housekeeping gene *Tbp*. *p<0.001 (Student's *t* test). B) Spry4 protein levels were analyzed by IB in Cos cells transfected with *Spry4* shRNA or a control vector together with Myc-Spry4. Actin is shown as loading control. C) Spry4 knockdown on Erk2/MAPK activation was analyzed in PC12 cells treated with NGF (50 ng/ml) for 10 min. Erk2/MAPK activation was evaluated by transient transfection of HA-tagged Erk2 plasmid with a control or *Spry4* shRNA construct into PC12 cells. Erk2 activation (P-Erk2) was evaluated by HA-immunoprecipitation (IP) followed by IB with a specific antibody that recognizes the phosphorylated forms of ERK/MAPK. Reprobing of the same blot with anti-HA antibody is shown. Numbers below the lanes are normalized to the levels of HA-Erk2. D) Morphological differentiation of PC12 cells transfected with scramble (Ctrl) or *Spry4* shRNA cloned in the retroviral vector pGFP-V-RS and treated with NGF (50 ng/ml) for 24 h. E) The histogram shows the quantification of the relative number of GFP positive neurite-bearing cells longer than 1 or 2 cell diameters in the different experimental conditions. The results are shown as averages SD of a representative experiment performed in quadruplicates. *p<0.001 (Student's *t* test).

An shRNA expression vector directed to the rat *Spry4* mRNA sequence significantly reduced endogenous *Spry4* mRNA expression (Fig. 7A). Furthermore, this Spry4 shRNA vector abolished the levels of ectopically expressed Spry4 protein in Cos cells (Fig. 7B). In agreement with a role of Spry4 in the negative control of TrkA-mediated downstream signaling, Spry4 knockdown resulted in a substantial increase of MAPK activation in response to NGF (Fig. 7C). Likewise, a significant potentiation of PC12 cell differentiation was observed in cells transfected with a rat *Spry4* shRNA expression vector and stimulated with NGF for 24 h (Fig. 7D and E). Remarkably, such levels of differentiation would not normally be observed in control PC12 cells until after 48–72 h of NGF treatment.

## Discussion

**Figure 8:**
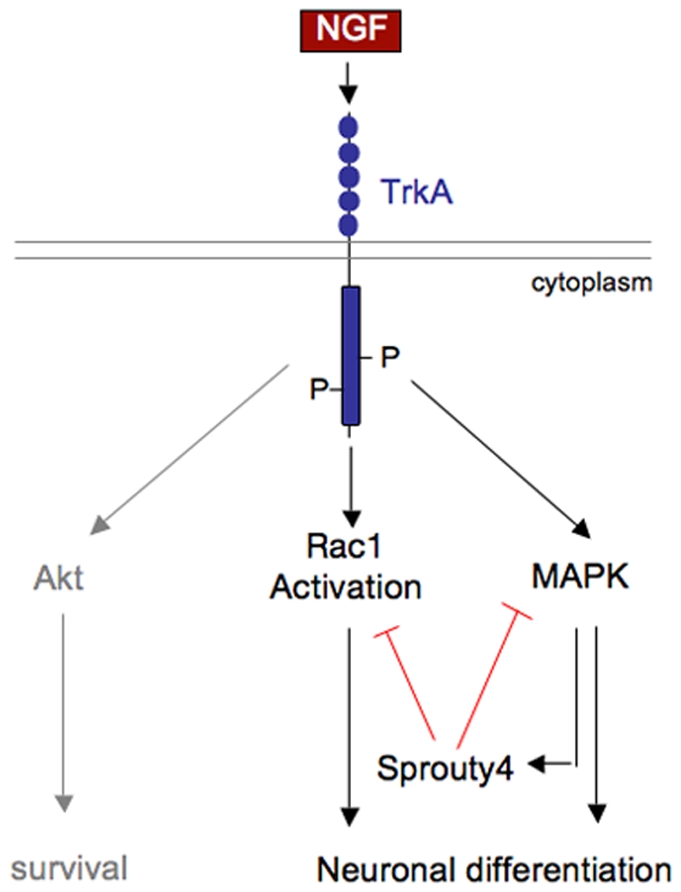
Model describing the role of Sprouty4 in NGF-induced TrkA receptor signaling and biology. NGF-TrkA signaling induces Sprouty4 expression via MAPK pathway. Then, Sprouty4 acting through a negative feedback loop specifically inhibits MAPK pathway and Rac1 activation, thereby preventing neuronal differentiation of PC12 and DRG neurons.

In this study, we provide evidence that Spry4, a NGF-regulated gene, restricts Erk/MAPK signaling and inhibits Rac1, but not Akt, activation in response to NGF. At molecular level, we identified the Spry4 Tyr-53 as an essential determinant for this inhibitory activity. We also found that Sprouty4 negatively regulates NGF-induced neuronal differentiation of PC12 cells and primary sensory neurons. Hence, we propose that Spry4 acts downstream of TrkA through a negative feedback loop to regulate neuronal differentiation by inhibiting Erk/MAPK signaling and Rac1 activation (Fig. 8).

PC12 cells and sensory neurons had provided to be good models to investigate NGF and FGF-induced neurite outgrowth. While in the present study, we demonstrate that endogenous Spry4 is a physiological negative feedback regulator of NGF-induced neuronal differentiation, previous studies proposed Spry2 and Spry4 as the critical regulators of Erk/MAPK activation related to neuronal cell differentiation in the context of FGF [12], [21].

High-affinity neurotrophin receptors, TrkA, TrkB and TrkC are expressed in distinct populations of DRG neurons where they control axonal development, target-innervation and neuronal survival mediated by NGF, BDNF and NT3, respectively. In particular, the NGF receptor, TrkA, is expressed in 80% of DRG neurons during the third embryonic week in rodents and their proportion drops to 40% postnatally [22]. Since our results indicate that Spry4 is involved in the differentiation of the TrkA-positive neuron subpopulation, it remains open the question whether other sprouty-family members could be modulating differentiation of other DRG neurons expressing TrkB or TrkC. The fact that Spry2 and Spry3 have been reported to control BDNF signaling in central nervous system neurons suggests that they could also have an analogous function modulating DRG neuronal differentiation by BDNF [8], [23].

Sprouty proteins have been shown to attenuate Erk/MAPK activation induced by a plethora of trophic factors, including FGF, HGF, VEGF, BDNF and GDNF. However, the molecular points at which Sprouty proteins disconnect RTK signaling pathways are still controversial [24]. Hence, even when many reports concluded that Spry may function downstream RTKs but upstream of Ras [10], [25]–[27], other studies demonstrated that Spry could inhibit RTK signaling at the level of Raf1 [28], [29].

In particular, mouse Spry4 inhibits VEGF-induced, Ras-independent ERK/MAPK activation at the level of Raf1, but has no effect on EGFR-induced, Ras-dependent activation of Raf1 [11], [30]. Previous studies demonstrated that NGF activates Erk/MAPK pathway through a Ras-dependent mechanism [31]. Here, using a constitutively active Ras construct (V12 Ras), we found that Spry4 was able to block Ras-dependent Erk/MAPK activation, indicating that in our system Spry4 acts downstream Ras, probably at the level of Raf1.

As it was previously observed with FGF and GDNF signaling [9], [12], overexpression of a dominant negative Spry4 Y53A mutant induced an increase in MAPK activation and cell differentiation in response to NGF (Fig. 6 B–D and Supplementary Fig. S3). Together, these dominant negative effects suggest that Spry4 mutant could be inhibiting other endogenous Sproutys forming homo-and/or hetero-dimers with them. Interestingly, clear differences in tyrosine phosphorylation of the Sprouty proteins by a given growth factor were observed. Thus, Spry1 was reported to be tyrosine phosphorylated by FGF and PDGF, Spry2 by FGF and EGF, whereas Spry4 was not tyrosine phosphorylated in response to any of these factors [20]. Consistent with previous data, we were not able to detect Spry4 tyrosine phosphorylation in response to NGF. According to this, our data demonstrate that Tyr-53 in itself, but not its phosphorylation is required for the inhibitory activity of Spry4 on NGF signaling.

The regulatory molecules that control the rate of neurite growth and the signals that determine when and where the axons and dendrites have to grow are still largely unknown. Interestingly, Gross *et. al* (2001) reported that overexpression of Spry1 and Spry2 in PC12 cells blocked neurite outgrowth and branching induced by NGF [10]. However, these authors failed to detect Spry1 and Spry2 in PC12 cells even after NGF treatment. This finding suggests that Spry1 and Spry2 are not physiologically involved in the morphological differentiation of PC12 cells. Therefore, it appears likely that other Sprouty family member may be up-regulated by NGF to control neurite outgrowth. Here, we characterize Spry4 as a physiological inhibitor of NGF-induced neurite elongation of PC12 cells and primary DRG neurons. Endogenous Spry4 expression was rapid and strongly up-regulated in PC12 cells and DRG neurons upon NGF treatment (Fig. 1). In contrast, we could not detect up-regulation of *Spry1* nor *Spry2* mRNAs upon NGF treatment.

Here, we demonstrate that Spry4, restricts neurite outgrowth induced by NGF without affecting survival. In agreement with this, Srpy4 was able to block Erk/MAPK and Rac1 but not Akt signaling pathway in response to NGF. In agreement with our data, various reports confirmed the role of other Sproutys in the control of neurite growth and branching induced by neurotrophic factors, such as FGF, BDNF and GDNF [8], [9], [12], [23]. In these studies, the authors have also linked the inhibitory effects of Spry2 to the Erk/MAPK pathway. However, Gross et al. have reported that in differentiated central nervous system neurons overexpression of Spry2 favored neuronal apoptosis, whereas its downregulation had a neuroprotective effect [8], suggesting that the apoptotic cell death might be due to the inhibition of a PI3K/Akt pathway, which is involved in neuronal survival. Edwin et al. reported that Spry2 requires the tumor suppressor phosphatase, PTEN, to inhibit Akt activation induced by the mitogen factor EGF. However, this inhibition of Akt activation by Spry2 is related to anti-proliferative and not to apoptotic effects. Thus, a clear link between apoptotic cell death and Akt inhibition by Spry2 has not been demonstrated yet. The fact that Spry2 antagonizes Akt activation and cell proliferation in response to EGF is consistent with the increase observed in the number of neurons produced by the loss of Spry in *Drosophila* [32]. More recently, Panagiotaki et al (2010) found that Spry3 also inhibits axonal morphogenesis *in vivo* and prevents filopodia formation in spinal cord neurons inhibiting Ca^2+^ signaling pathways activated by BDNF [23]. Remarkably, Spry3 acts as a very weak inhibitor of Erk/MAPK pathway downstream of BDNF/TrkB signaling. Thus, Sprouty proteins seem to behave as specific modulators of different RTK cascades and not as general inhibitors of RTK-induced MAPK pathway.

Neurite growth is an established marker of neuronal differentiation that requires the coordinated action of different signaling pathways. Consistent with this, NGF-induced neurite outgrowth have been reported to require a sustained MAPK activation as well as activation of Rac1-related pathways that play a key role regulating cytoskeletal changes. Here, we observed that Spry4 inhibition of TrkA signaling also induces a concomitant reduction of Rac1 activation. Together, our findings provide an insight into the mode of action of Spry on neurotrophin signaling and biology. Work from recent years has revealed that Spry targets are more diverse than originally assumed [11], [19], [23], [33], [34]. In this regards, the present inhibition of Rac1-related pathways by Spry4 also contribute to highlight the versatility of this Sprouty family member to regulate diverse trophic factor-induced signaling pathways.

Our findings indicate that by modulating Spry4 function, NGF and TrkA receptors can regulate Rac1 and Erk/MAPK signaling pathways required for neurite outgrowth. The accurate regulation of neurotrophin signaling is crucial for the normal development of the nervous system. In the developing nervous system, neurite outgrowth is an essential process underlying the formation of highly specific patterns of neuronal connectivity. Abnormalities in neurite formation and extension have been speculated to cause disorganization of neuronal network and eventual neural disorders. Hence, the abnormal expression of Sproutys in different neuronal types, blocking neurotrophic factor signaling, might be associated to neurological diseases [35]. Interestingly, mutations in the S*pred1* gene, which codify for a structural and functional protein related to Sprouty, has been identified in pathologies characterized by macroencephaly and mental retardation [36], [37].

Therefore, further characterization of the mechanisms through which Sprouty proteins are regulated in neurons may contribute to understand the underlying causes of many neurodegenerative diseases and neurological syndromes.

## Materials and Methods

### Ethics Statement

Animal experiments were approved by Stockholms Norra djurförsöksetiska nämnd (Dnr: 360/06) and by the institutional animal care and ethichs committee of the Shool of Medicine (CICUAL-UBA), ethical permit number: 25473/2010.

### Cell lines and recombinant proteins

Cos cells were grown in DMEM supplemented with 10% FBS [38] and neuroblast-like PC12 cells were grown in DMEM supplemented with 5% horse serum and 10% FBS [18]. NGF was purchased from Promega. PC12-Sprouty4 cells (clon S2) and PC12-Sprouty4-Y53A cells (clones S-Y53A-12 and S-Y53A-17) were generated by stable transfection of the rat pheochromocytome cell line with Myc-tagged mSprouty4 and Myc-tagged mSprouty4 Y53A mutant respectively. Pak-CRIB was produced and purified using E.coli BL21, as previously described [18].

### Real-Time PCR

The expression of *Spry1, Spry2, Spry4* and TATA box binding protein (*Tbp*) mRNAs were analyzed. Total RNA was isolated from PC12 cells and DRG neurons treated with NGF (50 ng/ml) for different periods of time using RNA-easy columns (Quiagen). cDNA was synthesized using Multiscribe reverse transcriptase and random hexamers (Applied Biosystems). The cDNA was amplified using the following primer sets: TATA box binding protein (*Tbp*): forward, 5′-GGG GAG CTG TGA TGT GAA GT-3′; reverse, 5′- CCA GGA AAT AAT TCT GGC TCA-3′; Rat *Spry1*: forward, 5′-AAC AGT GTG GCA AGT GCA AA-3′; reverse, 5′-CAC AGG TAT CTG GAG CAG CA-3′; Rat *Spry2*: forward, 5′-TGG CAA GTG CAA GTG TAA GG-3′; reverse, 5′-ACC ATC GCG TAC AAC AGT GA-3′; Rat *Spry4*: forward, 5′-GCC CAT TGA CCA GAT GAA GAC-3′; reverse, 5′-TCC AGT GGC TTA CAG TGG AC-3′.

Real-time PCR was performed using the SYBR Green qPCR SuperMix (Invitrogen) on an ABI7500 sequence detection system (Applied Biosystems), according to the manufacturer's instructions. Reactions were performed in 25 l volume. Nucleotides, Taq DNA polymerase, and buffer were included in the LightCycler-DNA master SYBR Green mix (Invitrogen).

### Cell Transfection, Plasmids and pharmacological treatments

Cos cells were transfected with Polyethylenimine-PEI (Polysciences). PC12 cells were transfected using Fugene-6 (Roche) following manufacturer's instructions. Transient transfection of primary dorsal root ganglion (DRG) neurons was performed using Lipofectamine 2000 (Invitrogen) in 500 l of DMEM:F12 serum-free medium containing 1 g of total plasmid DNA (0.2 g of GFP+0.8 g of full-length cDNA of mouse wt-Sprouty4, Y53A-Sprouty4 or control vector).

Plasmids encoding 6xMyc epitope tag wt-Sprouty4 and the dominant negative mutant (Y53A-Sprouty4) were kindly provided by Dr. Akihiko Yoshimura (Kyushu University, Fukuoka, Japan). Plasmid encoding HA epitope tag Erk2/MAP Kinase was generously provided by Dr. José María Rojas (Instituto de Salud Carlos III, Madrid, Spain). Plasmid encoding GFP was obtained from Clontech. The plasmid encoding the GST-tagged GTPase binding domain (GBD) of PAK (PAK-GBD) was kindly provided by Dr. Hollis T. Cline (Cold Spring Harbor Laboratory, Cold Spring Harbor, NY, USA). The plasmid encoding the constitutive active form of H-Ras (HA-Ras-V12) was kindly provided by Dr. White E. and Dr. Chen J. (The Cancer Institute of New Jersey, NJ, USA). Pretreatment with the specific MEK inhibitor PD98059 (50 M) was done for 30 min at 37°C before NGF stimulation.

### Sensory neuron cultures

Dorsal root ganglion (DRG) neurons, from embryonic day 14.5 (E14.5) Sprague Dawley rats (School of Pharmacy and Biochemistry, University of Buenos Aires) were prepared as previously described [39]. Briefly, the ganglia were dissociated with trypsin and collagenase, and seeded onto poly-ornithine and laminin coated plates. The neurons were maintained in DMEM:F12 medium supplemented with 2 mM glutamine, 0.35% bovine serum albumin, 60 ng/ml progesterone, 16 mg/ml putrescine, 400 ng/ml L-tyroxine, 38 ng/ml sodium selenite, 340 ng/ml triiodo-thyronine, 60 mg/ml penicillin, 100 mg/ml streptomycin and NGF (50 ng/ml).

### Immunoprecipitation and Western blotting

Cells were lysated at 4°C in buffer containing 0.5% Triton X-100, 1% -octylglucoside plus protease and phosophatase inhibitors. Protein lysates were clarified by centrifugation and analyzed by immunoprecipitation and Western blotting as previously described [38]. The blots were scanned in a Storm 845 PhosphorImager (GE Healthcare Life Sciences), and quantifications were done with ImageQuant software (GE Healthcare Life Sciences). Numbers below the lanes indicate fold of induction relative to control normalized to total levels of the target protein.

The antibodies were obtained from various sources as follows: anti-phosphotyrosine (p-Tyr), anti-Myc and anti-Sprouty4 (H-100) were from Santa Cruz Biotechnology, anti-Rac1 was from BD Biosciences Pharmingen, anti-TrkA was from RnD Systems, anti-HA antibodies were from Roche, anti-phospho MAPK and anti-Phospho Akt (Ser 473) were from New England Biolabs and anti-III-Tubulin was from Promega.

### Pull down assays for Rac1 activation

To detect active, GTP-bound Rac1, we performed the pull-down assay using GST-Pak-CRIB, which specifically binds to active Rac1 (GTP-bound form). The band intensity in the immunoblot was quantified, and the levels of Rac-GTP were normalized to the amount of total Rac1.

### Immunofluorescence

Dissociated DRG neurons obtained from E14.5 rat embryos were fixed with 4% PFA, blocked with 10% donkey serum and then incubated with polyclonal anti-TrkA (dilution 1/200, R&D systems), and anti-Sprouty4 (dilution 1/100, Santa Cruz) antibodies. Second antibodies were from Jackson ImmunoResearch. Photographs were obtained using an Olympus IX-81 inverted microscope.

### Neurite outgrowth assays

For PC12 cell differentiation assays, the cells were transfected with GFP, GFP plus Myc epitope-tag wt-Sprouty4 or GFP plus Myc-tag Y53A-Sprouty4 using Fugene-6 reagent in complete medium. The next day the cells were plated on poly-D-lysine coated coverslips in 24 multiwell plates and cultured in DMEM medium supplemented with NGF (50 ng/ml). After 72 h, the cells were fixed with 4% paraformaldheyde (PFA) and stained with anti-Myc epitope antibodies. The number of cells bearing neurites longer than 1.5 cell bodies was quantified relative to the total number of neurons counted in at least 10 random fields of three different wells in each experiment. PC12 cell differentiation was evaluated in three independent experiments. The pictures were obtained using an Olympus IX-81 inverted microscope.

Neurite outgrowth assays were performed in dissociated cultures of E14.5 rat DRG neurons. Primary cultures were prepared as previously described (see above). Neurons were transfected with GFP plus empty vector (control group) or GFP plus 6xMyc epitope tag wt-Sprouty4 and cultured in the presence of NGF (50 ng/ml) for 36 h. Then, cells were fixed with 4% PFA and stained with anti–tubulin to identify neuronal cells. Neuronal survival was evaluated using the nuclear stain 4′, 6′ –diamino-2-phenylindole (DAPI) (Invitrogen). GFP-positive neurons containing fragmented or condensed nuclear staining were scored as apoptotic cells and not computed in the differentiation assays. Quantification of neurite length was performed using NIH ImageJ software.

### RNAi-mediated knockdown assays

Rat *Sprouty4* shRNA (29 mer) and scrambled negative control plasmids were purchased from Origene (Rockville, MD, USA) and used according to the manufacturer's protocols. The retroviral vector pGFP-V-RS was used for expression of shRNA targeting rat Spry4. The sequence of the *Spry4* shRNA is a 5′-GTG CAA GGT ATC TTC TAC CAC TGT ACT AA-3′, and corresponds to nucleotides 610–638 of rat *Spry4*. The specificity of Spry4 knockdown was confirmed by real-time PCR and immunoblotting (IB).

## Supporting Information

Figure S1 **NGF induces** ***Sprouty4*** **mRNA expression in PC12 cells.** Semiquantitative RT-PCR analysis (27 cycles) shows the expression levels of *Sprouty4* mRNA at different time-points after NGF stimulation. The expression level of the housekeeping gene *Tbp* was evaluated as an internal control.
(TIF)

Figure S2 **Sprouty4 restricts Rac1 activation in response to NGF.** Rac1 activation (Rac1-GTP) was also evaluated by transient transfection of PC12 cells with a control (empty vector) or a Myc-tagged Sprouty4 plasmid. After 36 h, cells were serum-starved and stimulated with or without NGF (50 ng/ml) for 10 min. Rac1 activation was assessed by GST-Pak-GBD pull-down assay, followed by immunoblot (IB) with anti-Myc antibodies to detect transfected Rac1. The bottom panels show total Rac1 and Myc-tagged Sprouty4 levels present in cell lysates.
(TIF)

Figure S3 **Regulation of neuronal differentiation of PC12-Spry4 and PC12-Spry4 Y53A cell lines in response to NGF.** A) Photomicrographs show parental PC12 cells, clones overexpressing Myc-tagged Spry4-wt construct (PC12-Spry4 cell line, clon S2) and cells stably transfected to express Myc-tagged Sprouty4 Y53A mutant (PC12-Spry4-Y53A cell line, clon S-Y53A-17). After 72 h of NGF treatment (50 ng/ml), the cells were fixed and stained with anti-Myc antibodies. Nuclear staining (in blue) with DAPI is also shown. Arrowheads indicate neuronal cell bodies and arrows denote neurite tips. Scale bar: 10 m. B) Histogram shows quantification of the relative number of PC12 cells bearing neurites longer than 1.5 cell body diameters after 72 h of treatment with NGF. The results are presented as averages SD of a representative experiment performed in triplicate. *p<0.05 and **p<0.001 (ANOVA followed by Student Newman Keuls). The experiment was repeated two times with similar results.
(TIF)

Figure S4 **Rac1-DN mimics the effects of Spry4 on NGF-induced PC12 cell differentiation.** A) Photomicrographs show PC12 cells co-transfected with either control plasmid or dominant negative Rac1 (Rac1-DN) construct together with a plasmid encoding GFP. The cells were stained with DAPI and rhodamine-conjugated phalloidin. Scale bar represents 10 m. Arrows indicate cell bodies and arrowheads indicate neurite tips.B) The histogram shows the quantification of the relative number of cells bearing neurites longer than 1 cell body diameter after 72 h of treatment with NGF. The results are presented as average SD of a representative experiment performed in triplicate. *, p<0.001 (one-way ANOVA, followed by Student-Newman-keuls test). Similar results were obtained in two independent experiments.
(TIF)

Figure S5 **Sprouty4 is not tyrosine phosphorylated in response to NGF.** Left panel, Myc-Spry4 transfected PC12 cells were treated with NGF at the indicated time points and then, the cell extracts were immunoprecipitated (IP) with anti-Myc antibodies followed by immunoblot (IB) with antibodies against phosphotyrosine (p-Tyr). Reprobing of the same blot with anti-Myc antibodies is shown. The experiment was repeated three times with identical results. Right panel, the same cell extracts used for the analysis of Spry4 phosphorylation were re-precipitated using anti-TrkA antibodies followed by immunoblot (IB) with antibodies against phosphotyrosine (p-Tyr) as an internal control of NGF stimulation and phosphotyrosine detection.
(TIF)
