# Supplementary figures and images for: Sprouty4 Is an Endogenous Negative Modulator of TrkA Signaling and Neuronal Differentiation Induced by NGF
Source: PLoS One. 2012 Feb 23;7(2):e32087. doi: 10.1371/journal.pone.0032087 (PMC3285629; doi:10.1371/journal.pone.0032087)

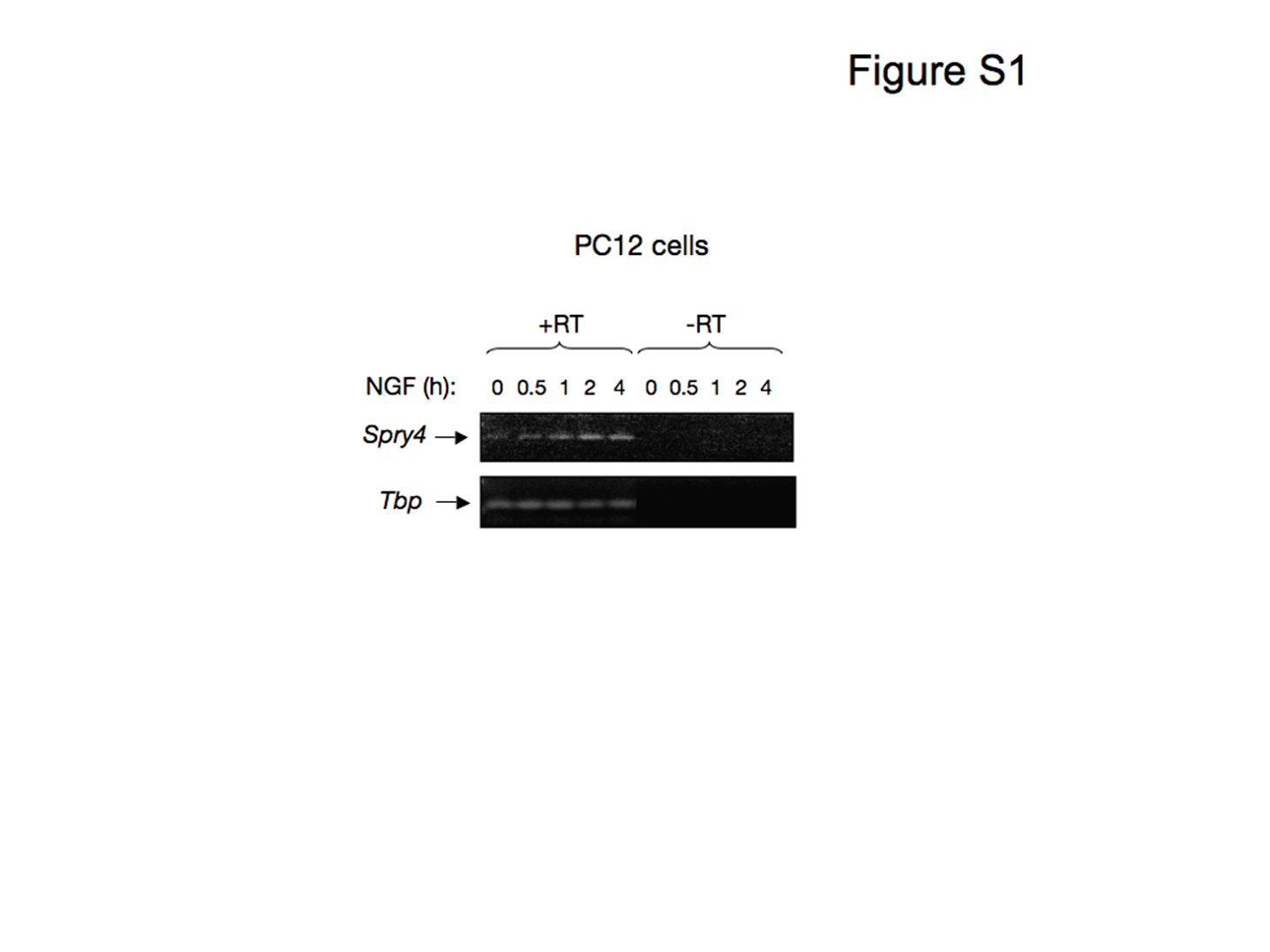

Supplement: Figure S1 — NGF induces Sprouty4 mRNA expression in PC12 cells. Semiquantitative RT-PCR analysis (27 cycles) shows the expression levels of Sprouty4 mRNA at different time-points after NGF stimulation. The expression level of the housekeeping gene Tbp was evaluated as an internal control. (TIF) [file pone.0032087.s001.tif]

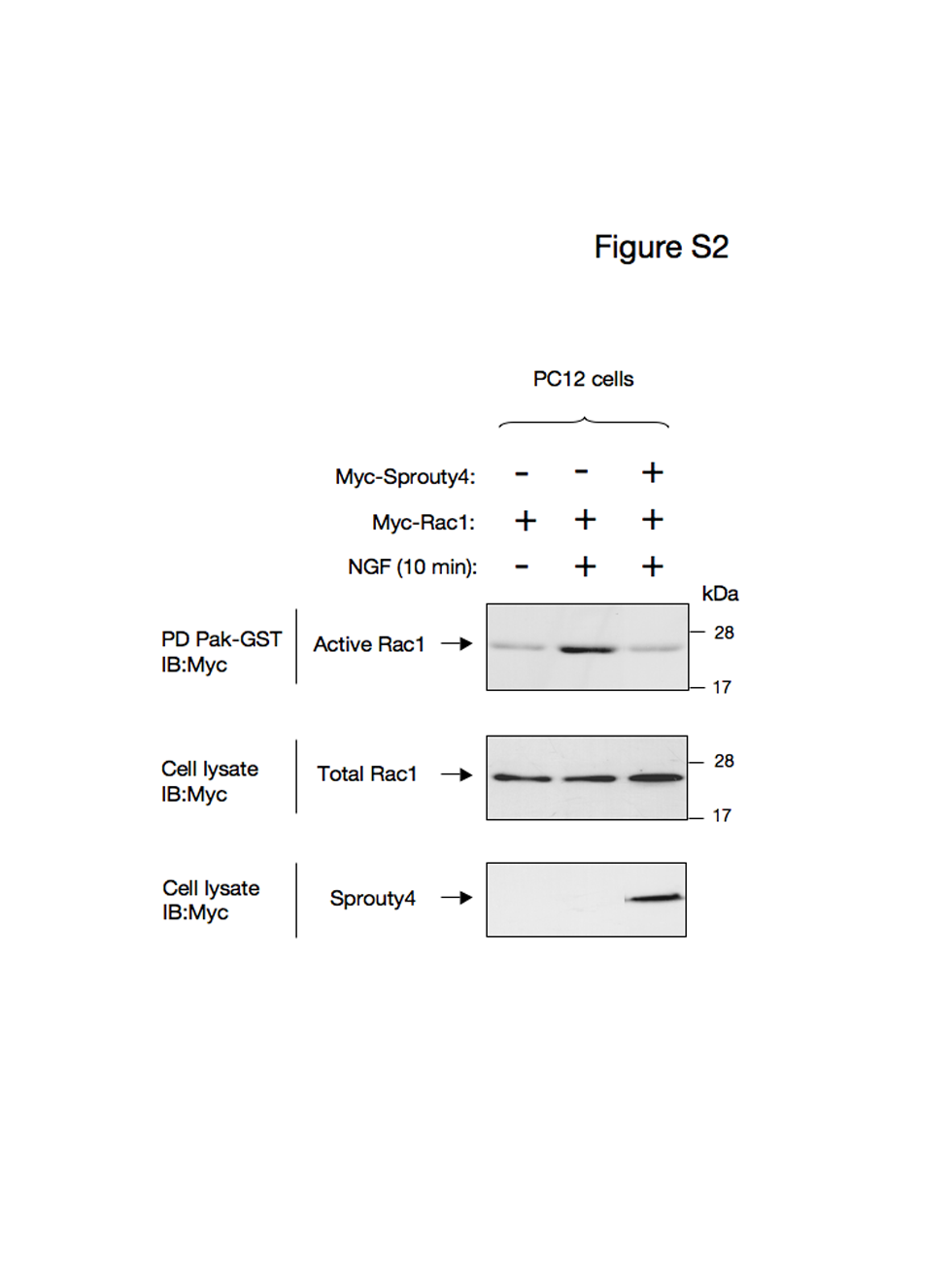

Supplement: Figure S2 — Sprouty4 restricts Rac1 activation in response to NGF. Rac1 activation (Rac1-GTP) was also evaluated by transient transfection of PC12 cells with a control (empty vector) or a Myc-tagged Sprouty4 plasmid. After 36 h, cells were serum-starved and stimulated with or without NGF (50 ng/ml) for 10 min. Rac1 activation was assessed by GST-Pak-GBD pull-down assay, followed by immunoblot (IB) with anti-Myc antibodies to detect transfected Rac1. The bottom panels show total Rac1 and Myc-tagged Sprouty4 levels present in cell lysates. (TIF) [file pone.0032087.s002.tif]

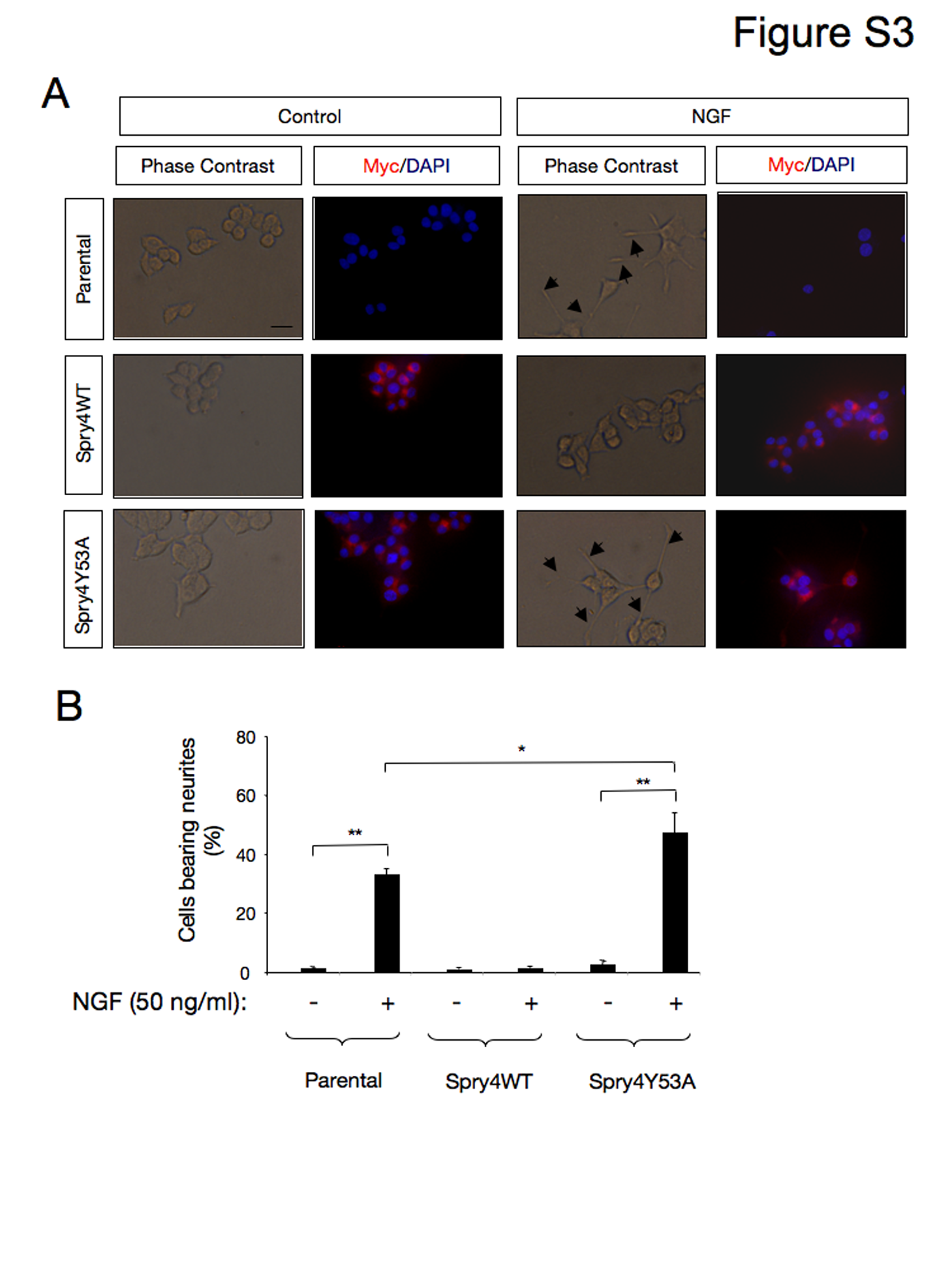

Supplement: Figure S3 — Regulation of neuronal differentiation of PC12-Spry4 and PC12-Spry4 Y53A cell lines in response to NGF. A) Photomicrographs show parental PC12 cells, clones overexpressing Myc-tagged Spry4-wt construct (PC12-Spry4 cell line, clon S2) and cells stably transfected to express Myc-tagged Sprouty4 Y53A mutant (PC12-Spry4-Y53A cell line, clon S-Y53A-17). After 72 h of NGF treatment (50 ng/ml), the cells were fixed and stained with anti-Myc antibodies. Nuclear staining (in blue) with DAPI is also shown. Arrowheads indicate neuronal cell bodies and arrows denote neurite tips. Scale bar: 10 m. B) Histogram shows quantification of the relative number of PC12 cells bearing neurites longer than 1.5 cell body diameters after 72 h of treatment with NGF. The results are presented as averages SD of a representative experiment performed in triplicate. *p<0.05 and **p<0.001 (ANOVA followed by Student Newman Keuls). The experiment was repeated two times with similar results. (TIF) [file pone.0032087.s003.tif]

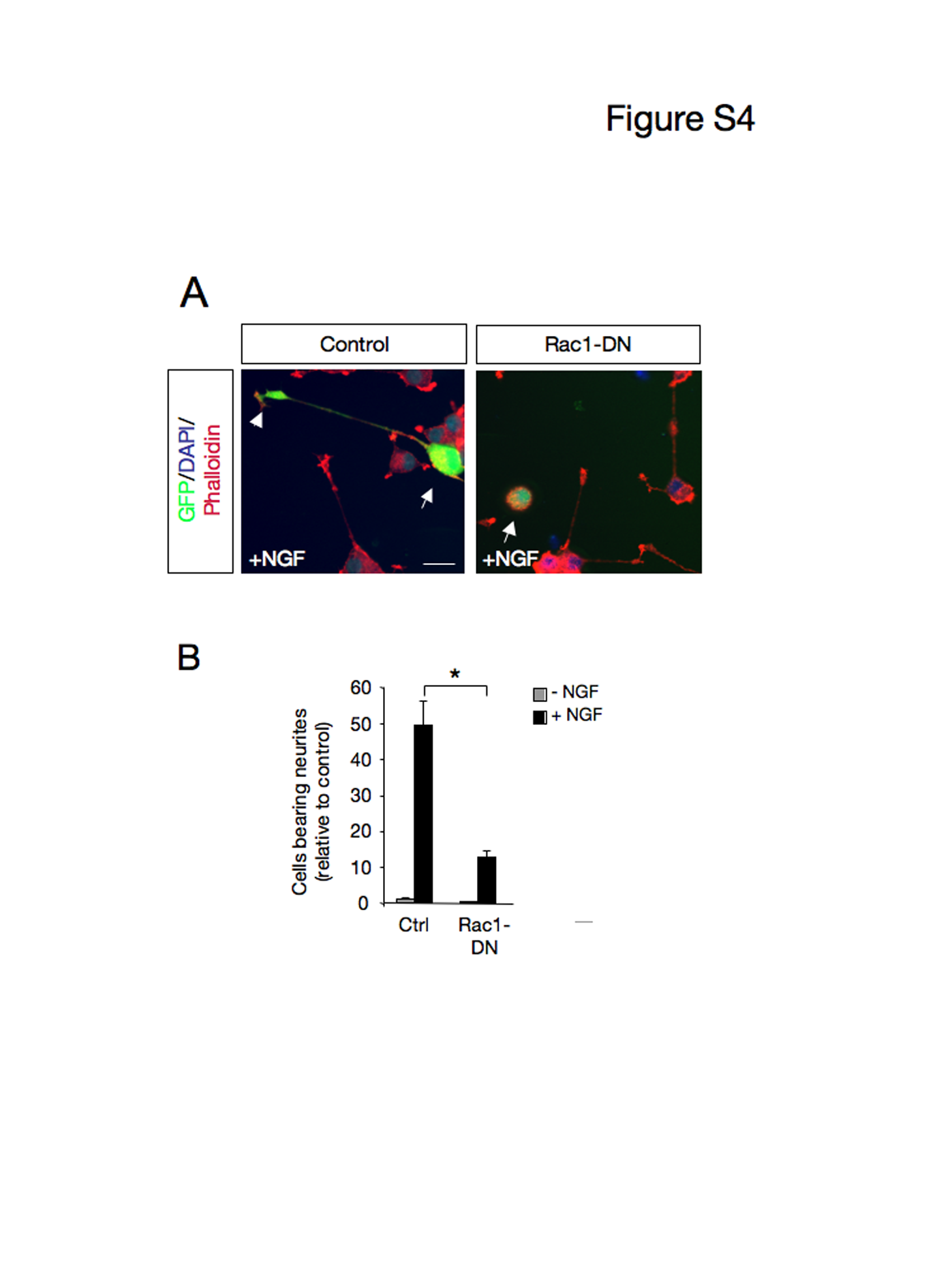

Supplement: Figure S4 — Rac1-DN mimics the effects of Spry4 on NGF-induced PC12 cell differentiation. A) Photomicrographs show PC12 cells co-transfected with either control plasmid or dominant negative Rac1 (Rac1-DN) construct together with a plasmid encoding GFP. The cells were stained with DAPI and rhodamine-conjugated phalloidin. Scale bar represents 10 m. Arrows indicate cell bodies and arrowheads indicate neurite tips.B) The histogram shows the quantification of the relative number of cells bearing neurites longer than 1 cell body diameter after 72 h of treatment with NGF. The results are presented as average SD of a representative experiment performed in triplicate. *, p<0.001 (one-way ANOVA, followed by Student-Newman-keuls test). Similar results were obtained in two independent experiments. (TIF) [file pone.0032087.s004.tif]

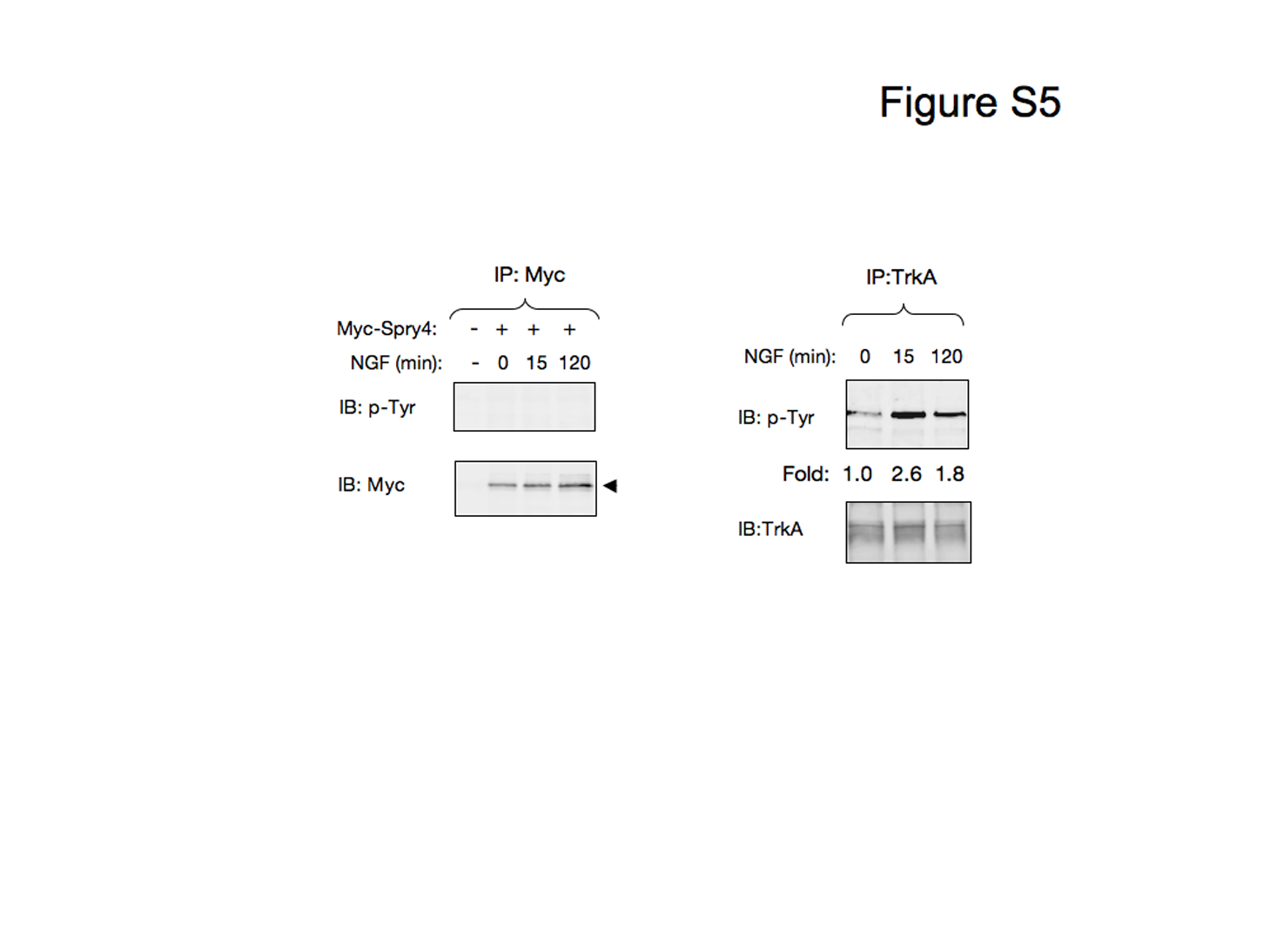

Supplement: Figure S5 — Sprouty4 is not tyrosine phosphorylated in response to NGF. Left panel, Myc-Spry4 transfected PC12 cells were treated with NGF at the indicated time points and then, the cell extracts were immunoprecipitated (IP) with anti-Myc antibodies followed by immunoblot (IB) with antibodies against phosphotyrosine (p-Tyr). Reprobing of the same blot with anti-Myc antibodies is shown. The experiment was repeated three times with identical results. Right panel, the same cell extracts used for the analysis of Spry4 phosphorylation were re-precipitated using anti-TrkA antibodies followed by immunoblot (IB) with antibodies against phosphotyrosine (p-Tyr) as an internal control of NGF stimulation and phosphotyrosine detection. (TIF) [file pone.0032087.s005.tif]
